# Genetic Evaluation of Natural Populations of the Endangered Conifer *Thuja koraiensis* Using Microsatellite Markers by Restriction-Associated DNA Sequencing

**DOI:** 10.3390/genes9040218

**Published:** 2018-04-17

**Authors:** Lu Hou, Yanhong Cui, Xiang Li, Wu Chen, Zhiyong Zhang, Xiaoming Pang, Yingyue Li

**Affiliations:** 1 National Engineering Laboratory for Tree Breeding, College of Biological Sciences and Technology, Beijing Forestry University, Beijing 100083, China; houlu822@163.com (L.H.); CUIYANHONG1314@163.com (Y.C.); lx2016bjfu@163.com (X.L.); xmpang@bjfu.edu.cn (X.P.); 2 The Germplasm Bank of Wild Species, Kunming Institute of Botany, Chinese Academy of Sciences, Kunming 650201, China; chenwu@mail.kib.ac.cn; 3 Beijing Key Laboratory of Ornamental Plants Germplasm Innovation and Molecular Breeding, National Engineering Research Center for Floriculture, Beijing Laboratory of Urban and Rural Ecological Environment, Key Laboratory of Genetics and Breeding in Forest Trees and Ornamental Plants of Ministry of Education, School of Landscape Architecture, Beijing Forestry University, Beijing 100083, China; zhangzhiyong543@163.com

**Keywords:** *Thuja koraiensis* Nakai, restriction-associated DNA (RAD) sequencing, simple sequence repeat (SSR) markers, population genetics, conservation genetics

## Abstract

*Thuja koraiensis* Nakai is an endangered conifer of high economic and ecological value in Jilin Province, China. However, studies on its population structure and conservation genetics have been limited by the lack of genomic data. Here, 37,761 microsatellites (simple sequence repeat, SSR) were detected based on 875,792 de novo-assembled contigs using a restriction-associated DNA (RAD) approach. Among these SSRs, 300 were randomly selected to test for polymorphisms and 96 obtained loci were able to amplify a fragment of expected size. Twelve polymorphic SSR markers were developed to analyze the genetic diversity and population structure of three natural populations. High genetic diversity (mean *N_A_* = 5.481, *H_E_* = 0.548) and moderate population differentiation (pairwise *F_st_* = 0.048–0.078, *N_m_* = 2.940–4.958) were found in this species. Molecular variance analysis suggested that most of the variation (83%) existed within populations. Combining the results of STRUCTURE, principal coordinate, and neighbor-joining analysis, the 232 individuals were divided into three genetic clusters that generally correlated with their geographical distributions. Finally, appropriate conservation strategies were proposed to protect this species. This study provides genetic information for the natural resource conservation and utilization of *T*. *koraiensis* and will facilitate further studies of the evolution and phylogeography of the species.

## 1. Introduction

*Thuja koraiensis* Nakai, an evergreen coniferous member of Cupressaceae, is mainly present in the Korean Peninsula and Changbai Mountain area of China [1]. A high seed abortion rate, low seed reproductive efficiency, and overexploitation have reduced the geographic distributions of natural *T*. *koraiensis* populations, and their natural regeneration is usually poor [2]. In 1992, the species was given third-class state protection in the Chinese Red Data Book [3] and was listed as a rare species by the World Conservation Monitoring Centre [4]. *T*. *koraiensis* has high adsorption ability of fluorine and therefore plays an important role in environmental protection [5]. Its wood has high density and oiliness, which results in it widely used in construction, handicrafts, and various other industries. Furthermore, its dried branches, leaves, and kernels are also used as traditional Chinese medicine, and have several biological activities [6].

Previous studies of *T*. *koraiensis* have focused mainly on its growth [5], morphological characteristics [1,7,8], and asexual reproduction [9], as well as the extraction and identification of aromatic substances [6,10,11]. The value of genetic information and genetic diversity analysis of endangered species has been demonstrated for breeding and genetic resource conservation programs [12,13]. Molecular markers have wide applications in linkage map construction, gene mapping and cloning, population genetic analysis, marker-assisted selection, and cultivar identification [14,15]. Among the various types of markers, microsatellites (simple sequence repeats, SSRs) are ideal for investigating population genetics because of their high numbers of polymorphisms, wide genomic distributions, codominant inheritance, and high degree of reproducibility [16,17,18]. To date, the population structures and conservation genetics of a large number of endangered tree species, such as *Tapiscia sinensis* [19], *Pulsatilla patens* [20], *Antirrhinum charidemi* [21], and *Origanum compactum* [22], have been studied using SSR markers. However, there are no SSR markers available for evaluating the genetic diversity and population structure of *T*. *koraiensis* and related species, making it difficult to implement feasible strategies for the conservation of this valuable endangered conifer tree.

The development of SSR markers for *T*. *koraiensis* has been hindered by a lack of genomic information, which are usually not available for rare and endangered species. The rapid developments of next-generation sequencing (NGS) technologies offer the possibility to generate large amounts of DNA sequences and develop molecular marker from nonmodel plants such as *Ziziphus jujube* [23], *Hevea brasiliensis* [24], and *Pistacia vera* [25]. Restriction-associated DNA (RAD) sequencing is one such NGS-based method which uses restriction enzymes on genomic DNA for complexity reduction [26,27]. Until now, RAD sequencing has been successfully applied for SSR discovery in *Solanum melongena* [28], *Sisymbrium austriacum* [29], *Gossypium hirsutum* [30], *Arachis hypogaea* [31], and *Lancea tibetica* [32]. Moreover, because of the conserved nature of protein-coding regions, RAD-derived SSRs have high polymorphism levels [33,34]. Thus, RAD sequencing technology is a powerful tool for mining microsatellites and developing markers quickly and more cost-effectively than other NGS methods [35].

The main aim of this study was to develop SSR markers based on RAD sequencing data, analyze the genetic diversity and population structure, and design conservation strategies for three natural populations including 232 individuals in northern China. This represents the first comprehensive description of the genetic diversity and population structure of existing natural *T*. *koraiensis* populations. The genetic resources and genic SSR markers collected will substantially contribute to further evolutionary and population genetic studies in *Thuja* and other related species.

## 2. Materials and Methods

### 2.1. Plant Material and DNA Extraction

**Figure 1:**
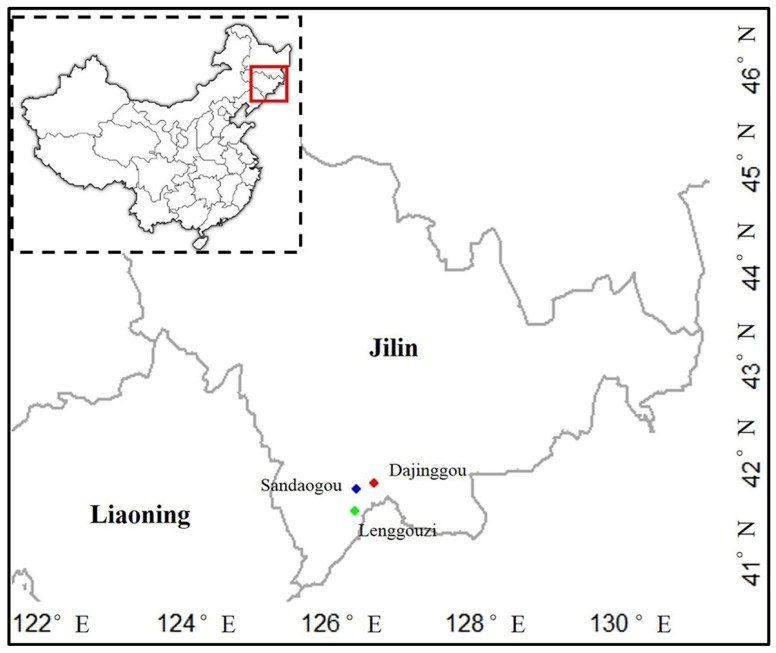
Geographic locations of the three natural populations of *Thuja koraiensis*. The map was created using the ‘maps’ and ‘mapdata’ package in R (v3.3.1, https://www.r-project.org/).

A field survey was conducted on the extant *T*. *koraiensis* populations from May to June 2016. Five extant natural forestry centers were investigated in Jilin Province, but *T*. *koraiensis* samples were found and collected in only three populations. In Lenggouzi (LGZ; 126°28′ E, 41°37′ N), more than 1000 trees were identified, and 155 samples were collected. Fifty individuals were found in Sandaogou (SDG; 126°28′ E, 41°51′ N), and 26 samples were collected. In Dajinggou (DJG; 126°44′ E, 41°52′ N), approximately 600 trees were identified, and 51 samples were collected (Figure 1, Table 1). To avoid sampling clones, the distances between sampled trees was ~50 m. Fresh leaves were dried in silica gel and stored at −80 °C until needed for DNA isolation. The genomic DNA for RAD sequencing was extracted from an adult tree using a Plant Genomic DNA Kit (Tiangen Biotech, Beijing, China) following the manufacturer’s procedures. Total genomic DNA of all samples was extracted by using the method of cetyltrimethylammonium bromide (CTAB) that was reported by Jin et al. [36]. The DNA was analyzed by agarose gel electrophoresis, and the concentration and purity were checked by a NanoDrop^®^ ND-1000 (Thermo Scientific, Wilmington, DE, USA) and Qubit 3.0 Fluorometer (Invitrogen, Carlsbad, CA, USA).

### 2.2. RAD Library Preparation, Sequencing, and Assembly

The DNA samples were processed into RAD libraries in a manner similar to that reported by Baird et al. [27]. Briefly, genomic DNA (1 µg) was digested for 60 min at 37 °C in a 20-µL reaction with 20 units (U) of *EcoRI*; then, the samples were heat-inactivated at 65 °C for 25 min. The P1 adapter was ligated to the products of the restriction reaction after digestion. One microliter of 10-µM P1 adapter was added to the sample, along with 1.5 µL T4 DNA Ligase and 2.5 µL 10× T4 DNA Ligase Buffer, and the reaction was incubated for 60 min at 4 °C, 15 min at 20 °C, and then held at 4 °C. Immediately after, DNA fragmentation was conducted; the VAHTS Fragmentase (Vazyme, Nanjing, China) was mixed thoroughly by gently vortexing for 3 s, and then the tube was transferred to ice. The fragmentation mix contained 25 µL DNA, 3 µL 10× VAHTS Fragmentase Reaction Buffer, and 2 µL VAHTS Fragmentase; and was incubated for 25 min at 37 °C and held at 4 °C. Samples were then separated by electrophoresis through a 2% agarose gel. The DNA fragments from 350 to 450 bp were isolated using a QIAquick Gel Extraction Kit (Qiagen, Hilden, Germany) and plused poly(A) tail. The following components were added to 40 µL of reaction liquid obtained from the previous step: 2 µL nuclease-free water, 5 µL End Repair Reaction Buffer (10×), and 3 µL End Prep Enzyme Mix, followed by incubation at 25 °C for 20 min and 72 °C for 20 min, and was then held at 4 °C. One microliter of 10 µM P2 adapter was ligated, as described above for P1. The ligated product was purified using 1.0× Agencourt AMPure XP Beads (Beckman Coulter Genomics, Danvers, MA, USA).

Polymerase chain reaction (PCR) enrichment of the library was performed in PCR reactions of 50-µL volume that contained 25 µL 2× Kapa HiFi PCR Mix (KAPA Biosystem, Wilmington, MA, USA), 1.25 µL Universal PCR Primer/i5 Prime, 1.25 µL Index (X) Primer, and 2.5 µL nuclease-free water. The PCR conditions were: initial denaturation at 98 °C for 45 s, then 15 cycles of 98 °C for 10 s, 60 °C for 30 s, and 72 °C for 30 s, and a final extension at 72 °C for 7 min. PCR products were purified using 1.0× Agencourt AMPure XP Beads following the manufacturer’s protocol, and then pooled together using the Qubit 3.0 Fluorometer (Invitrogen, Carlsbad, CA, USA) and Qsep100 Capillary electrophoresis apparatus (Bioptic, Taiwan, China). Sequencing was performed by Illumina Hiseq 2500 platform (Illumina, San Diego, CA, USA) that generated paired-end reads of 150 nucleotides. The preparation of DNA sequencing libraries and deep sequencing and analysis were performed by Beijing Ori-Gene Science and Technology Corp., Ltd. (Beijing, China). The raw data have been deposited in the NCBI Sequence Read Archive under the accession number SRR5338071.

### 2.3. RAD Sequence Analysis

For potential sequencing issues and contaminants, raw sequencing reads were filtered using FastQC (http://www.bioinformatics.bbsrc.ac.uk/projects/fastqc/). Adapter sequences, primers, Ns (unknown bases), short segments (<60 bp), and reads with quality scores below 30 were trimmed. The contigs were obtained using Trinity (http://trinityrnaseq.github.io/) and FLASH (http://www.cbcb.umd.edu/software/flash/) to splice high-quality sequences. Then, CD-HIT (https://github.com/weizhongli/cdhit) was used to cluster the sequences and remove the redundancy.

### 2.4. Sequence Annotation and Classification

For functional annotation of the de novo-assembled contigs, BLASTX alignment (E-value < 1.00 × 10^-5^) was performed against the public databases, including TrEMBL (http://www.ebi.ac.uk/uniprot), Swiss Institute of Bioinformatics databases (Swiss-Prot) (http://www.expasy.ch/sprot), protein families (Pfam) database (http://pfam.xfam.org/), NCBI nonredundant protein (Nr) database (http://www.ncbi.nlm.nih.gov), Cluster of Orthologous Groups for eukaryotic complete genomes (KOG) database (http://genome.jgi-psf.org/help/kogbrowser.jsf), and Kyoto Encyclopedia of Genes and Genomes (KEGG) pathway database (http://www.genome.jp/kegg). Based on the results of the Swiss-Prot and TrEMBL databases annotation, the gene ontology (GO) annotation of the contigs was obtained by Blast2GO [37].

### 2.5. SSR Identification and Marker Design

Sequences that contained SSR motifs were identified using the MISA search tool (http://pgrc.ipk-gatersleben.de/misa/). The parameters were set as follows: the minimum repeat unit was defined as 10 repeats for mononucleotide motifs; six repeats for dinucleotide motifs; and five repeats for tri-, tetra-, penta-, and hexanucleotide motifs. SSR primers were designed using Primer3 (https://sourceforge.net/projects/primer3/). Primers were designed according to the following criteria: amplified regions that ranged from 100–350 bp, primer annealing temperatures that ranged from 55–60 °C, optimal primer length that ranged from 18–24 bp, and GC content that ranged from 40–60%.

Furthermore, 300 SSR loci were randomly selected and screened in 15 individuals. Primers were synthesized by Sangon Biotech (Shanghai, China). These primers pairs were validated by PCR using the M13-tail technique, where an M13-tagged sequence (5′-TGTAAAACGACGGCCAGT-3′) was added at the 5′ end of all forward primers to allow fluorescent labeling (FAM, HEX, TAMRA, ROX). PCR amplifications, product separation, and scoring were performed as described [38].

### 2.6. Data Analysis

The genetic diversity indices, including the number of alleles (*N_A_*), effective number of alleles (*N_E_*), observed heterozygosity (*H_O_*), expected heterozygosity (*H_E_*), Shannon’s information index (I), and fixation index (F), were assessed at each locus and population levels using GeneAlEx version 6.5 [39]. The Hardy-Weinberg equilibrium (HWE) at each locus for each population was determined using GENEPOP version 4.2 [40,41]. The polymorphism information content (PIC) values were calculated using the program PIC_CALC version 0.6 [42]. To estimate the genetic variation among and within populations, the analysis of molecular variance (AMOVA) function of GeneAlEx version 6.5 [39] was performed. The average genetic differentiation index (*F_st_*) and gene flow (*N_m_*) were also calculated using GeneAlEx version 6.5 [39]. Gene flow estimation among the three populations was constructed using Migrate-n version 3.6.11 [43].

To examine the number of differentiated populations, STRUCTURE version 2.3.4 [44] based on a Bayesian analysis was run with K set values of 1 to 8. For each K, 10 independent runs were performed with a burn-in period of 100,000 iterations and a Markov chain Monte Carlo of 100,000 iterations. The most likely K was determined by combining two different approaches proposed by [44,45]. The results from STRUCTURE were processed with the software STRUCTURE HARVEST [46]. The principal coordinate analysis (PCoA) function of GeneAlEx version 6.5 [39] was used for further analysis of genetic structure. The genetic distances (GDs) as described by Nei et al. [47] between the individuals and populations were calculated using PowerMarker version 3.51 [48] and GeneAlex version 6.5 [39], respectively. Based on the resulting genetic distance matrix, a neighbor-joining (NJ) phylogenetic tree was constructed using MEGA version 6.0 [49] with 1000 bootstrap replicates.

## 3. Results

### 3.1. Sequencing, Contigs Assembly and Functional Annotation

Adult *T*. *koraiensis* tree from LGZ belonging to an existing natural population were used to construct a RAD library for sequencing (Figure 1). In total, 39.270 M raw reads were obtained from the RAD library. After quality filtering, 36.476 M (92.9%) clean reads were obtained. The Q20 and Q30 was greater than 96.4% and 91.9%, respectively, which indicates that data quality was very high (Table S1). The average GC content was 41.38% and the reads were assembled into 875,792 contigs with an average length of 262 bp (N50 = 274 bp) (Table 2).

**Figure 2:**
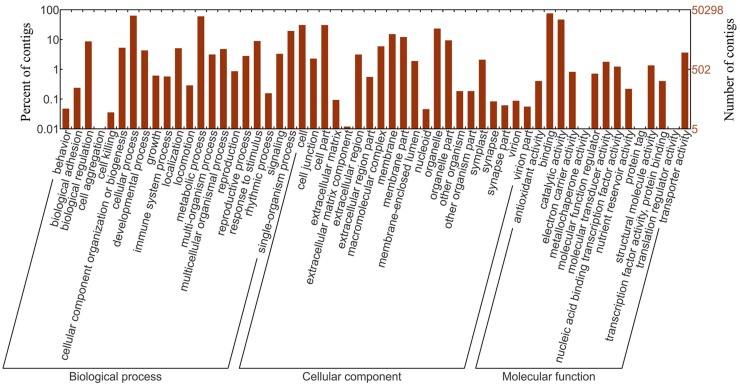
Gene ontology (GO) classification of *T*. *koraiensis* contigs.

Functional annotation of the contigs in *T*. *koraiensis* was searched against seven public databases. The BLASTX search against the Nr protein database revealed that 61.5% of contigs were present in unknown species, and the second-top hit species was *Vitis vinifera*, which accounted for 11.3% of the identified contigs (Figure S1). Among the *T*. *koraiensis* contigs, 50,298 contigs were assigned to one or more GO terms. There were 96,568 contigs categorized under biological processes; 69,335 under cellular component and 66,749 under molecular function (Figure 2). A total of 26,674 (3.0%) contigs were assigned to 25 KOG classifications. The cluster for general function prediction only represented the largest cluster (12,138 contigs), which is only related to basic metabolic and physiological functions, and only a few contigs were assigned to other clusters (Table S2). In the KEGG pathway-based analysis, 6805 contigs had significant matches and were assigned to 353 biological pathways. Of these, the spliceosome (ko03040, 233 contigs, 1.73%) pathway contained the largest number of contigs, followed by carbon metabolism (ko01200, 229 contigs, 1.70%) and protein processing in endoplasmic reticulum (ko04141, 210 contigs, 1.56%) (Table S3).

### 3.2. Frequency and Distribution of SSR Markers

A total of 875,792 sequences from *T*. *koraiensis* were searched for the presence of SSRs, and 30,102 sequences (3.44%) that contained 37,761 SSR markers were identified. Mononucleotide repeats were the most abundant, and accounted for 16,802 sequences (44.50%), followed by dinucleotide (14,795; 39.18%) and trinucleotide (5119; 13.56%) motifs (Table 3). The frequency of SSRs with different repeats numbers was also calculated. SSRs with 10 tandem repeats (7949; 21.05%) were most common, followed by those with six (4604; 12.19%), 11 (3957; 10.48%), five (3017; 7.99%), and seven tandem repeats (2720; 7.20%) (Table S4). The most abundant type was A/T (14,301; 37.87%), followed by AT/AT (6349; 16.81%), AC/GT (4776; 12.65%), AG/CT (3520; 9.32%), C/G (2501; 6.62%), ATC/ATG (1263; 3.35%), AAG/CTT (983; 2.60%), and AAT/ATT (768; 2.03%) motifs. The remaining motifs accounted for only 8.74%. The AT/AT and ATC/ATG repeats were the most dominant di- and trinucleotide motifs, respectively. Furthermore, there were very few CCG/CGG (57; 0.15%) and no CG/GC motifs in *T*. *koraiensis* (Table S5).

### 3.3. Development, Validation and Polymorphism of SSR Markers

SSR loci with mononucleotide motifs were excluded from primer design, because they easily lead to high mismatch ratios during DNA amplification [50]. A total of 899 primer pairs were designed by Primer3. In this study, we randomly selected and tested 300 primer pairs, of which 96 yielded fragments of expected sizes and showed stable amplification (Table S6), and the remaining primer pairs failed to yield any products. As assessed by capillary electrophoresis, 84 (28%) of the SSR markers were monomorphic or had very low levels of polymorphism that exhibited clear, single peaks for each allele, and 12 (4%) were polymorphic for multiple alleles. The allelic PIC values were 0.124–0.798, with six of 12 possessing highly informative scores (PIC > 0.50), five having moderately informative scores (0.50 > PIC > 0.25), and one possessing a weakly informative score (0. 25 > PIC > 0). The average PIC value was 0.492. Markers BFTK-273 and BFTK-194 had the highest and lowest genetic diversity, respectively, with 0.820 and 0.133 for *H_E_* values, respectively, and 0.798 and 0.124 for PIC values, respectively. Ten of the 12 polymorphic markers showed HWE, while the others showed significant deviations from HWE after a Bonferroni correction, which was probably because of small sample size and heterozygote deficiency (Table 4).

### 3.4. Population Genetic Diversity and Differentiation

In the three natural populations of *T*. *koraiensis*, the genetic diversity parameter *N_A_* per population varied from 3.667 (SDG) to 7.667 (LGZ), with a mean of 5.481, and the *N_E_* varied from 2.235 (DJG) to 2.750 (SDG), with a mean of 2.514. The average *H_O_* and *H_E_* values were 0.644 and 0.548, respectively, and ranged from 0.537 (DJG) to 0.766 (LGZ) and from 0.491 (DJG) to 0.582 (SDG), respectively. The I values, indicative of genetic diversity, had an average of 1.008. The average value of F was −0.128 and ranged from −0.036 in the DJG population to −0.279 in the LGZ population (Table 5). These parameters suggested a relatively high genetic diversity within and among *T*. *koraiensis* populations. DJG had the lowest values for *H_E_*, I, and PIC, suggesting that individuals from DJG had lower genetic diversity than those in the LGZ and SDG populations.

**Figure 3:**
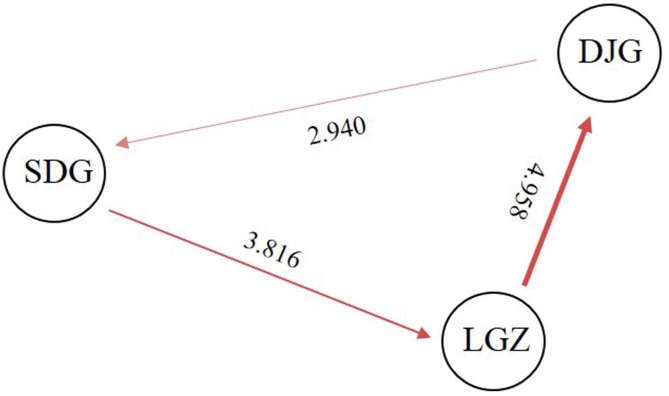
Estimations of gene flow among three populations by Migrate-n [43]. The width of the line and the number shown next to the arrows indicate the gene flow (*N_m_*) values. LGZ: Lenggouzi; SDG: Sandaogou; DJG: Dajinggou.

The AMOVA analysis revealed that 17% of the total variance occurred among the populations and that 83% was due to variance within three populations (Table 6), indicating that a higher variation level resided within populations than among populations of *T*. *koraiensis*. The *F_ST_* and *N_m_* values were almost congruent with the AMOVA results. The highest *F_ST_* value observed was only 0.078 between SDG and DJG, while the lowest, between LGZ and DJG, was 0.048 (Table 7). The lowest *N_m_* value was 2.940 and was observed between SDG and DJG, while the highest was 4.958 and observed between LGZ and DJG (Figure 3).

### 3.5. Population Genetic Structure

**Figure 4:**
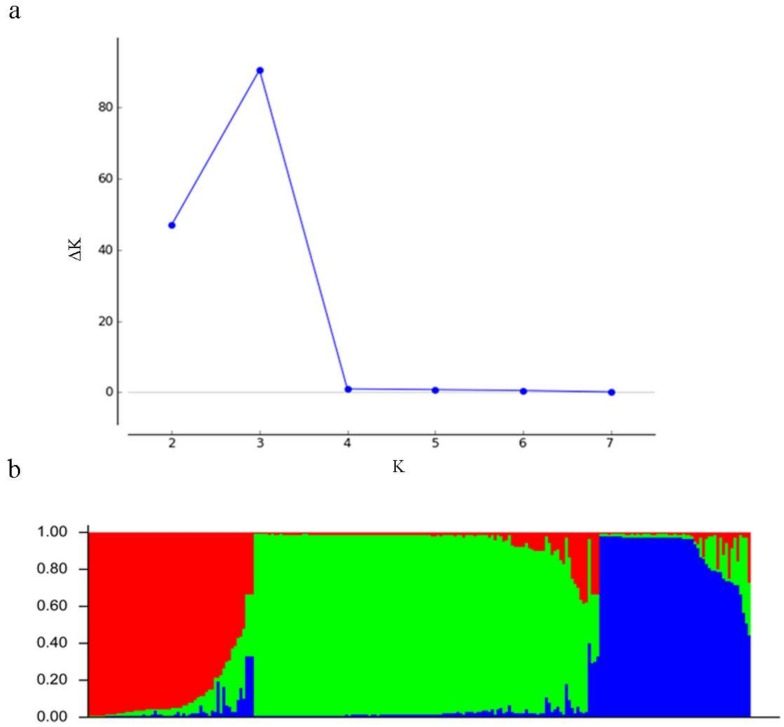
Results of STRUCTURE analysis based on microsatellite data: (**a**) Estimation of population using LnP(D)-derived ΔK with cluster number (K) ranged from 1 to 8; (**b**) estimated genetic structure of the three populations based on STRUCTURE analysis with K = 3.

Analysis with STRUCTURE HARVESTER software showed that ΔK is largest at K = 3, and second largest at K = 2 (Figure 4a). Furthermore, Bayesian methods implemented in STRUCTURE were used to analyze the genetic structure of 232 *T*. *koraiensis* individuals (Figure 4b). The individual with the probability higher than a score of 0.80 was considered as a pure one, and lower than 0.80 as an admixture one. The clusters labeled in red, green, and blue included 55 (45 pure and 10 admixture), 124 (109 pure and 15 admixture), and 53 (39 pure and 14 admixture) individuals, respectively. The individuals from the LGZ population almost entirely made up the green cluster. Similarly, most individuals from the SDG (14 of 26) and DJG (43 of 51) population were assigned to the red and blue clusters, respectively. The 232 individuals could be divided into three clusters corresponding to their geographical distributions.

**Figure 5:**
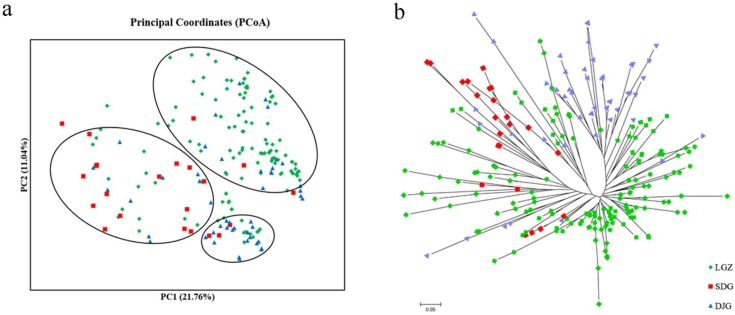
Principal coordinate analysis (PCoA) (**a**) and neighbour-joining (NJ) tree (**b**) of the 232 *T*. *koraiensis* individuals. PC1: the first principal coordinate; PC2: the second principal coordinate.

To further evaluate the population structure, the PCoA was conducted. The first two principal coordinates explained 21.76% and 11.04% respectively, and explained 32.8% of the total variation (Figure 5a). An NJ phylogenetic tree was constructed based on a matrix of GDs among individuals (Figure 5b). The PCoA and NJ tree separated the sampled individual trees into three clusters corresponding to their geographical origins, although slight admixed features were observed in each cluster, which was consistent with the STRUCTURE analysis. The GDs of the populations were also calculated and ranged from 0.135 (between LGZ and DJG populations) to 0.233 (between SDG and DJG populations) (Table S7).

## 4. Discussion

To establish conservation strategies, a good understanding of the genetic diversity and population structure of endangered species is the necessary first step towards a long-term goal of sustainability. However, research into *T*. *koraiensis* is still in its early stages, and genetic studies are very limited. For these reasons, using a RAD approach, we performed genome assembly of *T*. *koraiensis* for the development of SSR markers that were also employed to determine the genetic diversity in extant natural populations.

In this study, the sequence assembly generated 875,792 contigs with an average length of 262 bp (N50 = 274 bp). The number of contigs for *T*. *koraiensis* was higher than other plants in assembly analysis of RAD sequencing studies such as *Cynara cardunculus* [51], *Helianthus annuus* [52], *Arundinaria faberi* [53], and *L*. *tibetica* [32], suggesting that there were abundant *EcoR*I restriction sites in the *T*. *koraiensis* genome. The sequencing depth, assembly method, and large genome may have contributed to larger numbers of assembled contigs [30]. The N50 and average length of assembled contigs are longer than in other species such as *A*. *faberi* (N50 = 246 bp, average contig length = 240 bp), *Yushania brevipaniculata* (N50 = 249 bp, average contig length = 240 bp) [53], and *Epimedium sagittatum* (average contig length = 224.9 bp) [54]. In the *T*. *koraiensis* assembly, the total number, N50 value and average length of contigs are all superior to other RAD-sequenced species, which indicates better quality contig assembly [55]. The GC dinucleotide content for *T*. *koraiensis* assembled contigs was 41.38%, which is consistent with results from RAD sequencing studies in *A*. *faberi* (44%) [53] and *S*. *austriacum* (41.5%) [29]; somewhat higher than that in *Arabidopsis thaliana* (36%, TAIR 10 genome database), *V*. *vinifera* [56], *C*. *cardunculus* (37.4%) [51], and *H*. *annuus* (36.2%) [52], although the enzyme *EcoR*I (GAATTC) with rich AT may bias the dataset towards low GC content. These genome sequences will provide important information for developing molecular markers and conducting genome research on *T*. *koraiensis*.

The functional annotation of *T*. *koraiensis* contigs was searched against seven public databases. In the GO analysis, biological process was the most significantly enriched GO term, comprising 50,298 annotations, and metabolic process (30,758) was prominent, indicating that these are involved in important metabolic activities. The conifer species, including *T*. *koraiensis*, are adversity resistant, especially in cold resistance, but the mechanisms underlying this adaptation are unknown. In the KEGG pathway annotation, 1133 contigs were identified in signal transduction, such as ATP-binding cassette transporters (ABC transporters) and cyclic adenosine monophosphate (cAMP) and adenosine 5′-monophosphate (AMP)-activated protein kinase (AMPK) signaling pathways, which are relevant to stress responses. Among the environmental adaptation pathways, the circadian clock plays important roles in activating the adaptation of a species to the local environment by regulating physiological activities [57]. In total, 214 contigs were assigned to environmental adaptation, and 24 contigs were involved in circadian rhythm. These functional annotation results are in agreement with other studies in conifers, such as *Sabina chinensis* [55] and *Platycladus orientalis* [58]. They will make a valuable contribution to further studies of biological pathways, functions, structures, and interactions of specific genes in *T*. *koraiensis*.

Based on these contigs, we successfully developed 37,761 SSR loci. The frequency of RAD-derived SSR occurrence in *T*. *koraiensis* is higher than those observed in *S*. *austriacum* [29], *G*. *hirsutum* [30], and *A*. *hypogaea* (2.9%) [31], which indicates that the RAD technique is an effective method for discovering genome-wide SSRs in *T*. *koraiensis* and closely related species. In previous studies, di- or trinucleotide motifs were identified as most prevalent for most organisms [59], but there are exceptions [60,61,62]. The distribution of SSRs in *T*. *koraiensis* revealed that mononucleotide motifs were the most abundant (44.50%), followed by dinucleotide (39.18%) and trinucleotide (13.56%) motifs. These results agree with other observations in *Prunus armeniaca* [63], *A*. *thaliana*, and *Populus trichocarpa* [64]. The most abundant dinucleotide motif is consistent with *G*. *hirsutum* and *S*. *melongena* (AT/AT), but the most abundant trinucleotide motif is different from these species (ATC/ATG *T*. *koraiensis*, for AAT/ATT for *G*. *hirsutum*) [28,30]). It is interesting that there were no CG/GC repeat motifs and very few CCG/CGG repeats in *T*. *koraiensis*, which strongly supports previous studies which indicated that CG/GC and CCG/CGG repeats are very infrequent in a large number of dicotyledonous plants, but the most predominant in monocots [54,65,66]. Among the 16,802 mononucleotide SSRs detected, 14,301 were A/T; these loci have been suggested to fill gaps in linkage maps constructed with higher order SSRs [65]. The frequency and distribution patterns vary depending on many factors including mining tool used, size of sequence dataset, and SSR identification criteria applied, whereas SSR abundance greatly depends on plant species [31,67].

A relatively high genetic diversity was found using the SSR marker analysis in 232 *T*. *koraiensis* individuals from three natural populations, with mean *N_A_* and *H_E_* values of 5.481 and 0.548, respectively. The genetic diversity of *T*. *koraiensis* was slightly lower than that of conifer species that have widespread geographic ranges, including *Thuja occidentalis* (mean *N_A_* = 8.83; *H_E_* = 0.64 in the core populations; mean *N_A_* = 6.64; *H_E_* = 0.60 in the peripheral populations) [68], *Pinus densiflora* (mean *N_A_* =14.6; *H_E_* = 0.873 within 1883 individuals from 62 natural populations) [69], and *P*. *orientalis* (mean *N_A_* = 8.945; *H_E_* = 0.832 from 21 populations) [70]. Compared with relatively abundant species, especially their widespread congeners, rare species, endemic plants and endangered species usually exhibit lower genetic diversity [71,72]. However, the values determined here represented higher SSR-derived genetic diversity than in other endangered endemic perennial species, such as *Paeonia jishanensis* (mean *N_A_* = 2.376; *H_E_* = 0.340 within 236 individuals from 10 extant populations) [73], *Taxus wallichiana* (mean *N_A_* = 4.154; *H_E_* = 0.538 within 130 individuals from 13 geographically separate populations) [74], and *Pinus dabeshanensis* (mean *N_A_* = 3.700; *H_E_* = 0.360 within 148 samples from four extant populations) [75]. The average value of *H_O_* (0.644) was slightly greater than that of *H_E_* (0.548), and the value of F was negative at the population level, indicating that excess heterozygosity was existed within the entire natural distribution range of the species.

Moderate population differentiation (pairwise *F_st_* = 0.048–0.078) and weak population structure were found among the three natural populations, which may be subject to local habitat conditions. The AMOVA analysis revealed that most (83%) of the total molecular variance was within-population, which are similar to results obtained for other Cupressaceae species including *Taxodium distichum* (81.47%) [76], *T*. *occidentalis* (88%) [68], and *Juniperus thurifera* (90.50%) [77], based on SSR markers. However, the extent of the population differentiation within *T*. *koraiensis* is much greater than that within *P*. *orientalis*, in which the genetic variation was only 1.25% among populations, with greatest variation among individuals within populations and within individuals (98.75%) [36]. This variation difference may be explained by the larger population size and wider distribution of *P*. *orientalis* compared with those of *T*. *koraiensis*. The genetic differentiation into distinct populations is strongly influenced by genetic drift, gene flow, long-term evolution, mating systems, selection, and mutations [78,79]. In this study, the *N_m_* value among *T*. *koraiensis* populations ranged from 2.940 to 4.958, indicating the frequent flow of genes and continuous distribution of populations. The population structure analysis divided the three populations into three clusters that were significantly related to their geographical origins, and some trees from the populations had a mixed ancestry, especially in the LGZ and SDG populations. According to botanical characteristics, *T*. *koraiensis* is wind-pollinated and its pollen can spread over long distances, which may explain the high level of genetic diversity. The studied trees of *T*. *koraiensis* were sampled within a 220-km radius of Jilin Province. The relatively small geographic range and anemophilous pollination could produce more frequent gene exchange events and result in little differentiation between populations.

The main goal of conservation is to develop a suitable strategy for maintaining current genetic diversity and ensuring the long-term evolution of an endangered species [80,81]. In the field survey, the numbers of adult trees in the three studied populations were all small, with only 35 adult individuals observed in the LGZ population. Many seedlings of different ages and a number of immature individuals were present in three extant natural forestry centers. In many areas, including the Sanchazi and Xiatianping natural forestry centers, naturally occurring individuals were reportedly present in 2015 [82]. However, no additional individuals were found during this survey (personal observation). We hypothesize that the considerable reduction in population size and the current narrow distribution may be the result of habitat loss and excessive deforestation. The SDG and DJG populations, which exhibited small population sizes and lower genetic diversity, are susceptible to anthropogenic interference. Thus, a conservation strategy should be prioritized for in situ conservation, including protecting as many populations and individuals as possible to avoid further losses of genetic variation and the use of cuttage seedlings to increase the population sizes. In addition, the combination of in situ and ex situ conservation approaches can be critical for safeguarding valuable genetic resources [83]. For the LGZ population, which has the most individuals and the highest genetic diversity, a germplasm resource repository could be established by transplantation and artificial breeding. The three extant natural populations of *T*. *koraiensis* of LGZ, SDG, and DJG should serve as a valuable baseline for future monitoring of the effectiveness of a conservation strategy to maintain and restore genetic diversity in this conifer.

## Figures and Tables

**Table 1:** Location and sampling site characteristics for the three *Thuja koraiensis* populations.

Populations	Location	Number	Longitude (E)	Latitude (N)	Altitude/m
LGZ	Lenggouzi, Changbai, Jilin	155	126°28′	41°37′	1108
SDG	Sandaogou, Baishan, Jilin	26	126°28′	41°51′	483
DJG	Dajinggou, Baishan, Jilin	51	126°44′	41°52′	1172

LGZ: Lenggouzi; SDG: Sandaogou; DJG: Dajinggou.

**Table 2:** Summary statistics of the restriction-associated DNA (RAD) tags sequencing.

Feature	Value
Number of contigs	875,792
No. of contigs (≥200 bp)	805,284
No. of contigs (≥500 bp)	2227
No. of contigs (≥1000 bp)	95
N50 (bp)	274
GC (%)	41.38
Total contig length (bp)	230,095,060
Max. contig length (bp)	4265
Min. contig length (bp)	150
Average contig length (bp)	262

N50: weighted median statistic such that 50% of the entire assembly is contained in contigs equal to or greater than this value.

**Table 3:** Summary of simple sequence repeats (SSRs) identified in *T*. *koraiensis*.

Searching Item	Number
Sequences examined	875,792
Size of examined sequences (bp)	230,095,060
Identified SSRs	37,761
SSR-containing sequences	30,102
Sequences containing more than one SSR	5212
SSRs present in compound formation	7253
Mononucleotide SSRs	16,802
Dinucleotide SSRs	14,795
Trinucleotide SSRs	5119
Tetranucleotide SSRs	478
Pentanucleotide SSRs	133
Hexanucleotide SSRs	434

**Table 4:** Characterization of 12 polymorphic SSRs.

SSR Name	Contigs	Primer Sequence (5′–>3′)	Motif	Tm (°C)	Size (bp)	*N_A_*	*H_O_*	*H_E_*	PIC	HWE
BFTK-39	contig_120265	F: TGTTCACTCCTCATCCACCG R: ACCGACATGATCTGCACACA	(TG)_11_	60	200	4	1.000	0.653	0.599	ns
BFTK-51	contig_169311	F: TCATTGGAGTTGTATGGTGTCA R: TGCACAATTTGACCACTTGGA	(CT)_34_	59	159	3	0.571	0.561	0.465	ns
BFTK-68	contig_220530	F: ACAACAAAGCGGTGGTAAACC R: GAATTGATGCTCAGCAGCCG	(GA)_14_	60	198	3	0.375	0.461	0.398	ns
BFTK-123	contig_387595	F: TGCTTGCACTTGGATGTTGTG R: GCTCGATGCCAGGGTTTTTC	(TG)_19_	60	175	3	0.800	0.540	0.466	ns
BFTK-136	contig_412632	F: CCCCCGGGCATAGATCAAAT R: CCCCCGGGCATAGATCAAAT	(TA)_10_	60	183	3	1.000	0.594	0.511	ns
BFTK-167	contig_487894	F: TGAAGTCCCCATCTACATGTCA R: CTCAAACCAACTCCGTTACCT	(TG)_16_	59	169	3	0.125	0.664	0.590	**
BFTK-187	contig_551058	F: AGGACACAGAACAGAGCAGC R: CGGGTTAGCACATCAGGGAT	(AAC)_10_	60	147	2	0.750	0.469	0.359	ns
BFTK-194	contig_576296	F: TACCTCGGAGATCAACCCCA R: CTCCCTCACATGGATGCCAA	(GA)_13_	60	109	2	0.143	0.133	0.124	ns
BFTK-199	contig_589312	F: ATAGGGCACGACTAGCTTGC R: CATTCTTCAGCCTCCTGGTGT	(AC)_15_	60	132	4	0.667	0.667	0.620	ns
BFTK-261	contig_680582	F: AGAGGTGGGGAAGAGGAGAC R: AGGCCCTAAACCCTATAACCA	(AG)_9_	60	142	5	0.286	0.653	0.602	*
BFTK-273	contig_689976	F: TCCCATGTTTGTGGTCTCAGT R: TCCCCCAGAGTGCAACATTC	(TTG)_9_	60	209	7	1.000	0.820	0.798	ns
BFTK-295	contig_111296	F: CGCAAGTCCAAATCAGCAAC R: TCGTGCAAACTTCCGTACCA	(CAA)_6_	59	146	2	0.750	0.500	0.375	ns
Mean						3.417	0.622	0.559	0.492	

Tm: annealing temperature; *N_A_:* number of alleles; *H_O_:* observed heterozygosity; *H_E_:* expected heterozygosity; PIC: polymorphism information content; HWE: Hardy–Weinberg equilibrium; ns: not significant; * *p* < 0.05; ** *p* < 0.01.

**Table 5:** Genetic diversity of the three populations of *T*. *koraiensis*.

Populations	*N_A_*	*N_E_*	I	*H_O_*	*H_E_*	F	PIC
LGZ	7.667	2.557	1.091	0.766	0.572	−0.279	0.503
SDG	3.667	2.750	1.024	0.629	0.582	−0.068	0.504
DJG	5.111	2.235	0.908	0.537	0.491	−0.036	0.428
Mean	5.481	2.514	1.008	0.644	0.548	−0.128	0.478

*N_E_*: effective number of alleles; I: Shannon’s information index; F: fixation index.

**Table 6:** Results of the analysis of molecular variance (AMOVA) for three populations of *T*. *koraiensis*.

Source	df	SS	MS	Percentage of Variation (%)
Among Populations	2	164.500	82.250	17%
Within Populations	229	1489.435	6.504	83%
Total	231	1653.935	88.754	100%

df: degrees of freedom; SS: sum of squares; MS: mean of squares.

**Table 7:** Pairwise genetic differentiation index (*F_st_*) values between the three populations.

Populations	LGZ	SDG	DJG
LGZ			
SDG	0.061		
DJG	0.048	0.078	

## Supplementary Materials

The following are available online at http://www.mdpi.com/2073-4425/9/4/218/s1, Figure S1: Distribution of the top BLASTX hits for the contigs in the NCBI nonredundant protein (Nr) database. Table S1: Summary data of read using FastQC after quality control. Table S2: The Cluster of Orthologous Groups for eukaryotic complete genomes (KOG) annotation. Table S3: The Kyoto Encyclopedia of Genes and Genomes (KEGG) annotation. Table S4: Frequencies of repeat type with repeat numbers in SSRs from *T*. *koraiensis*. Table S5: Frequencies of different repeat motifs in SSRs. Table S6: The list of 96 SSRs with stable amplification. Table S7: Nei’s genetic distance (below diagonal) and genetic identity (above diagonal) among the three populations.

## References

[1] Sun B., Cui Y.M., Wang H.F., Ferguson D.K., Xiang Q.P., Ma Q.W., Wang Y.F. (2015). Recognizing the species of *Thuja* (Cupressaceae) based on their cone and foliage morphology. Phytotaxa.

[2] Yin H., Jin H., Zhao Y., Qin L.W., Dai Y.H., Liu L., Zhao W. (2016). Present situation and conservation strategy of rare and endangered species *Thuja koraiensis* in Changbai mountain. J. Beihua Univ..

[3] Fu L.G. (1992). China Plant Red Data Book.

[4] He X., Lin J., Hu Y., Wang X., Li F. (1996). Comparison among threatened categories of conifers from China. Chin. Biodivers..

[5] Liu C., Wang Y., Shi S. (2009). Height growth regularity of *Thuja koraiensis*. Jilin For. Sci. Technol..

[6] Chung I.M., Praveen N., Ahmad A. (2011). Composition of the essential oil and antioxidant activity of petroleum ether extract of *Thuja koraiensis*. Asian J. Chem..

[7] Tang X., Li Z.Y., Hu Y.X. (2005). Wood anatomy of *Thuja sutchuenensis* endemic to China. J. Wuhan Bot. Res..

[8] Wang H.S., Deng Z.G., Huang Z.Z., Li R.X. (2007). Structural research on secondary xylem of stem of *Thuja koraiensis* Nakai under SEM. J. Tonghua Norm. Univ..

[9] Yin H., Zhao Y., Cui K., Liu L.J., Yu C., Chen Q., Dai Y., Zhao W. (2013). Asexual reproduction technique of *Thuja koraiensis* Nakai. Chin. Wild Plant Resour..

[10] Yang Z., Tian Z., Liu Q., Sun Y. (1994). Studies on the chemical constituents of the volatile oil from leaves of *Thuja Koraiensis* Nakai maxim. J. Northeast Norm. Univ..

[11] Qi J.Z., Sun G.R., Yang W.S., Sun R.C., Xue F. (1995). Analysis on the chemical constituents of essential oil from branches and leaves of *Thuja Koraiensis* Nakai. J. Plant Resour. Environ..

[12] Cohen J.I., Williams J.T., Plucknett D.L., Shands H. (1991). Ex situ conservation of plant genetic resources: Global development and environmental concerns. Science.

[13] Ouborg N.J. (2010). Integrating population genetics and conservation biology in the era of genomics. Biol. Lett..

[14] Aranzana M., Carbó J., Arús P. (2003). Microsatellite variability in peach [*Prunus persica* (L.) Batsch]: Cultivar identification, marker mutation, pedigree inferences and population structure. Theor. Appl. Genet..

[15] Rajaram V., Nepolean T., Senthilvel S., Varshney R.K., Vadez V., Srivastava R.K., Shah T.M., Supriya A., Kumar S., Kumari B.R. (2013). Pearl millet (*Pennisetum glaucum* (L.) R. Br.) consensus linkage map constructed using four RIL mapping populations and newly developed EST-SSRs. BMC Genom..

[16] Kijas J.M.H., Fowler J.C.S., Thomas M.R. (1995). An evaluation of sequence tagged microsatellite site markers for genetic analysis within Citrus and related species. Genome.

[17] Powell W., Morgante M., Andre C., Hanafey M., Vogel J., Tingey S., Rafalski A. (1996). The comparison of RFLP, RAPD, AFLP and SSR (microsatellite) markers for germplasm analysis. Mol. Breed..

[18] Du Q., Wang B., Wei Z., Zhang D., Li B. (2012). Genetic diversity and population structure of Chinese white poplar (*Populus tomentosa*) revealed by SSR markers. J. Hered..

[19] Zhou X.J., Ren X.L., Liu W.Z. (2016). Genetic diversity of SSR markers in wild populations of *Tapiscia sinensis*, an endangered tree species. Biochem. Syst. Ecol..

[20] Szczecińska M., Sramko G., Wołosz K., Sawicki J. (2016). Genetic diversity and population structure of the rare and endangered plant species *Pulsatilla patens* (L.) Mill in East Central Europe. PLoS ONE.

[21] Forrest A., Escudero M., Heuertz M., Wilson Y., Cano E., Vargas P. (2017). Testing the hypothesis of low genetic diversity and population structure in narrow endemic species: The endangered *Antirrhinum charidemi* (Plantaginaceae). Bot. J. Linn. Soc..

[22] Aboukhalid K., Machon N., Lambourdière J., Abdelkrim J., Bakha M., Douaik A., Korbecka-Glinkaf G., Gabouna F., Tomig F., Lamirib A. (2017). Analysis of genetic diversity and population structure of the endangered *Origanum compactum* from Morocco, using SSR markers: Implication for conservation. Biol. Conserv..

[23] Ma Q., Feng K., Yang W., Chen Y., Yu F., Yin T. (2014). Identification and characterization of nucleotide variations in the genome of *Ziziphus jujuba* (Rhamnaceae) by next generation sequencing. Mol. Biol. Rep..

[24] De Souza L.M., Toledo-Silva G., Cardoso-Silva C.B., Da Silva C.C., de Araujo Andreotti I.A., Conson A.R.O., Mantello C.C., Guen V., de Souza A.P. (2016). Development of single nucleotide polymorphism markers in the large and complex rubber tree genome using next-generation sequence data. Mol. Breed..

[25] Motalebipour E.Z., Kafkas S., Khodaeiaminjan M., Çoban N., Gözel H. (2016). Genome survey of pistachio (*Pistacia vera* L.) by next generation sequencing: Development of novel SSR markers and genetic diversity in Pistacia species. BMC Genom..

[26] Miller M.R., Dunham J.P., Amores A., Cresko W.A., Johnson E.A. (2007). Rapid and cost-effective polymorphism identification and genotyping using restriction site associated DNA (RAD) markers. Genome Res..

[27] Baird N.A., Etter P.D., Atwood T.S., Currey M.C., Shiver A.L., Lewis Z.A., Selker E.U., Cresko W.A., Johnson E.A. (2008). Rapid SNP discovery and genetic mapping using sequenced RAD markers. PLoS ONE.

[28] Barchi L., Lanteri S., Portis E., Acquadro A., Valè G., Toppino L., Rotino G.L. (2011). Identification of SNP and SSR markers in eggplant using RAD tag sequencing. BMC Genom..

[29] Vandepitte K., Honnay O., Mergeay J., Breyne P., Roldán-Ruiz I., De Meyer T. (2013). SNP discovery using Paired-End RAD-tag sequencing on pooled genomic DNA of *Sisymbrium austriacum* (Brassicaceae). Mol. Ecol. Resour..

[30] Wang H., Jin X., Zhang B., Shen C., Lin Z. (2015). Enrichment of an intraspecific genetic map of upland cotton by developing markers using parental RAD sequencing. DNA Res..

[31] Gupta S.K., Baek J., Carrasquilla-Garcia N., Penmetsa R.V. (2015). Genome-wide polymorphism detection in peanut using next-generation restriction-site-associated DNA (RAD) sequencing. Mol. Breed..

[32] Tian Z., Zhang F., Liu H., Gao Q., Chen S. (2016). Development of SSR markers for a Tibetan medicinal plant, *Lancea tibetica* (Phrymaceae), based on RAD sequencing. Appl. Plant Sci..

[33] Durand J., Bodénès C., Chancerel E., Frigerio J.M., Vendramin G., Sebastiani F., Buonamici A., Gailing O., Koelewijn H.P., Villani F. (2010). A fast and cost-effective approach to develop and map EST-SSR markers: Oak as a case study. BMC Genom..

[34] Lesser M.R., Parchman T.L., Buerkle C. (2012). Cross-species transferability of SSR loci developed from transcriptome sequencing in lodgepole pine. Mol. Ecol. Resour..

[35] Chutimanitsakun Y., Nipper R.W., Cuesta-Marcos A., Cistué L., Corey A., Filichkina T., Johnson E.A., Hayes P.M. (2011). Construction and application for QTL analysis of a Restriction Site Associated DNA (RAD) linkage map in barley. BMC Genom..

[36] Jin Y.J., Ma Y.P., Wang S., Hu X.G., Huang L.S., Li Y., Wang X.R., Mao J.F. (2016). Genetic evaluation of the breeding population of a valuable reforestation conifer *Platycladus orientalis* (Cupressaceae). Sci. Rep..

[37] Conesa A., Götz S., García-Gómez J.M., Terol J., Talón M., Robles M. (2005). Blast2GO: A universal tool for annotation, visualization and analysis in functional genomics research. Bioinformatics.

[38] Zhang Z.Y., Cui B.B., Mao J.F., Pang X.M., Li Y.Y. (2015). Novel polymorphic EST-derived microsatellite markers for the red-listed five needle pine, *Pinus dabeshanensis*. Conserv. Genet. Resour..

[39] Peakall R., Smouse P.E. (2012). GenAlEx 6.5: Genetic analysis in Excel. Population genetic software for teaching and research—An update. Bioinformatics.

[40] Raymond M., Rousset F. (1995). GENEPOP (version 1.2): Population genetics software for exact tests and ecumenicism. J. Hered..

[41] Rousset F. (2008). GENEPOP’007: A complete reimplementation of the GENEPOP software for Windows and Linux. Mol. Ecol. Resour..

[42] Liu H.Y., Li C.Y., Xiong F. (2008). Isolation and characterization of 19 polymorphic microsatellite loci from *Neosalanx taihuensis*, a rapidly invasive and adaptative species. Biochem. Syst. Ecol..

[43] Beerli P., Palczewski M. (2010). Unified framework to evaluate Panmixia and migration direction among multiple sampling locations. Genetics.

[44] Pritchard J.K., Stephens M., Donnelly P. (2000). Inference of population structure using multilocus genotype data. Genetics.

[45] Evanno G., Regnaut S., Goudet J. (2005). Detecting the number of clusters of individuals using the software STRUCTURE: A simulation study. Mol. Ecol..

[46] Earl D.A., vonHoldt B.M. (2012). STRUCTURE HARVESTER: A website and program for visualizing STRUCTURE output and implementing the Evanno method. Conserv. Genet. Resour..

[47] Nei M., Tajima F., Tateno Y. (1983). Accuracy of estimated phylogenetic trees from molecular data. J. Mol. Evol..

[48] Liu K., Muse S. (2004). PowerMarker: New genetic data analysis software. Version 3.23. Bioinformatics.

[49] Tamura K., Stecher G., Peterson D., Filipski A., Kumar S. (2013). MEGA6: Molecular evolutionary genetics analysis version 6.0. Mol. Biol. Evol..

[50] Cheng Y., Yang Y., Wang Z., Qi B., Yin Y., Li H. (2015). Development and characterization of EST-SSR markers in *Taxodium* ‘*zhongshansa*’. Plant Mol. Biol. Report..

[51] Scaglione D., Acquadro A., Portis E., Tirone M., Knapp S.J., Lanteri S. (2012). RAD tag sequencing as a source of SNP markers in *Cynara cardunculus* L.. BMC Genom..

[52] Pegadaraju V., Nipper R., Hulke B., Qi L., Schultz Q. (2013). De novo sequencing of sunflower genome for SNP discovery using RAD (restriction site associated DNA) approach. BMC Genom..

[53] Wang X.Q., Zhao L., Eaton D.A.R., Li D.Z., Guo Z.H. (2013). Identification of SNP markers for inferring phylogeny in temperate bamboos (Poaceae: Bambusoideae) using RAD sequencing. Mol. Ecol. Resour..

[54] Zeng S., Xiao G., Guo J., Fei Z., Xu Y., Roe B.A., Wang Y. (2010). Development of a EST dataset and characterization of EST-SSRs in a traditional Chinese medicinal plant, *Epimedium sagittatum* (Sieb. Et Zucc.) Maxim. BMC Genom..

[55] Hu X.G., Liu H., Zhang J.Q., Sun Y.Q., Jin Y.Q., Zhao W., El-Kassaby Y.A., Wang X.R., Mao J.F. (2016). Global transcriptome analysis of *Sabina chinensis* (Cupressaceae), a valuable reforestation conifer. Mol. Breed..

[56] Jaillon O., Aury J., Noel B., Policriti A., Clepet C., Casagrande A., Choisne N., Aubourg S., Vitulo N., Jubin C. (2007). The grapevine genome sequence suggests ancestral hexaploidization in major angiosperm phyla. Nature.

[57] Niwa Y., Yamashino T., Mizuno T. (2009). Circadian clock regulates photoperiodic response of hypocotyl elongation through a coincidence mechanism in *Arabidopsis thaliana*. Plant Cell Physiol..

[58] Hu X.G., Liu H., Jin Y., Sun Y.Q., Li Y., Zhao W., El-Kassaby Y.A., Wang X.R., Mao J.F. (2016). De novo transcriptome assembly and characterization for the widespread and stress-tolerant conifer *Platycladus orientalis*. PLoS ONE.

[59] Zhang Q., Ma B., Li H., Chang Y., Han Y., Li J., Wei G., Zhao S., Khan M., Zhou Y. (2012). Identification, characterization, and utilization of genome-wide simple sequence repeats to identify a QTL for acidity in apple. BMC Genom..

[60] Cavagnaro P.F., Senalik D.A., Yang L.M., Simon P.W., Harkins T.T., Kodira C.D., Huang S., Weng Y. (2010). Genome-wide characterization of simple sequence repeats in cucumber (*Cucumis sativus* L.). BMC Genom..

[61] Zou C., Lu C., Zhang Y., Song G. (2012). Distribution and characterization of simple sequence repeats in *Gossypium raimondii* genome. Bioinformation.

[62] Wang L.X., Elbaidouri M., Abernathy B., Chen H.L., Wang S.H., Lee S.H., Jackson S.A., Cheng X.Z. (2015). Distribution and analysis of SSR in mung bean (*Vigna radiata,* L.) genome based on an SSR-enriched library. Mol. Breed..

[63] Kumpatla S.P. (2004). Computational Mining and Survey of Simple Sequence Repeats (SSRs) in Expressed Sequence Tags (ESTs) of Dicotyledonous Plants. Master’s Thesis.

[64] Sonah H., Deshmukh R.K., Sharma A., Singh V.P., Gupta D.K., Gacche R.N., Rana J.C., Singh N.K., Sharma T.R. (2011). Genome-wide distribution and organization of microsatellites in plants: An insight into marker development in *Brachypodium*. PLoS ONE.

[65] Kumpatla S., Mukhopadhyay S. (2005). Mining and survey of simple sequence repeats in expressed sequence tags of dicotyledonous species. Genome.

[66] Jia H., Yang H., Sun P., Li J., Zhang J., Guo Y., Han X., Zhang G., Lu M., Hu J. (2016). De novo transcriptome assembly, development of EST-SSR markers and population genetic analyses for the desert biomass willow, *Salix psammophila*. Sci. Rep..

[67] Tóth G., Gáspári Z., Jurka J. (2000). Microsatellites in different eukaryotic genome, survey and analysis. Genome Res..

[68] Pandey M., Rajora O.P. (2012). Genetic diversity and differentiation of core vs. peripheral populations of eastern white cedar, *Thuja occidentalis* (Cupressaceae). Am. J. Bot..

[69] Iwaizumi M.G., Tsuda Y., Ohtani M., Tsumura Y., Takahashi M. (2013). Recent distribution changes affect geographic clines in genetic diversity and structure of *Pinus densiflora* natural populations in Japan. For. Ecol. Manag..

[70] Lin Z., Lou A. (2013). Old-Growth *Platycladus orientalis* as a resource for reproductive capacity and genetic diversity. Plos ONE.

[71] Huh M., Huh H.W. (1999). Patterns of genetic diversity and population structure of the clonal herb, *Potentilla fragarioides* var. *sprengeliana* (Rosaceae) in Korea. Acta Bot. Sin..

[72] Cole C.T. (2003). Genetic variation in rare and common plants. Annu. Rev. Ecol. Evol. Syst..

[73] Xu X.X., Cheng F.Y., Xian H.L., Peng L.P. (2003). Genetic diversity and population structure of endangered endemic *Paeonia jishanensis* in China and conservation implications. Biochem. Syst. Ecol..

[74] Zhang D.Q., Zhou N. (2003). Genetic diversity and population structure of the endangered conifer *Taxus wallichiana* var. *mairei* (Taxaceae) revealed by Simple Sequence Repeat (SSR) markers. Biochem. Syst. Ecol..

[75] Zhang Z.Y., Wang H., Chen W., Pang X.M., Li Y.Y. (2003). Genetic diversity and structure of native and non-native populations of the endangered plant *Pinus dabeshanensis*. Genet. Mol. Res..

[76] Tanaka A., Ohtani M., Suyama Y., Inomata N., Tsumura Y., Middleton B.A., Tachida H., Kusumi J. (2012). Population genetic structure of a widespread coniferous tree, *Taxodium distichum*, [L.] Rich. (Cupressaceae), in the Mississippi River Alluvial Valley and Florida. Tree Genet. Gen..

[77] Teixeira H., Nabais C. (2014). Genetic diversity and differentiation of *Juniperus thurifera* in Spain and Morocco as determined by SSR. PLoS ONE.

[78] Hamrick J.L., Godt M. (2014). Effects of life history traits on genetic diversity in plant species. Philos. Trans. R. Soc. Lond. B Biol. Sci..

[79] Schaal B.A., Hayworth D.A., Olsen K.M., Rauscher J.T., Smith W.A. (1998). Phylogeographic studies in plants: Problems and prospects. Mol. Ecol..

[80] Forest F., Grenyer R., Rouget M., Davies T.J., Cowling R.M., Faith D.P., Balmford A., Manning J.C., Proches S., van der Bank M. (1998). Preserving the evolutionary potential of floras in biodiversity hotspots. Nature.

[81] Rodrigues L., Berg C.V.D., Povoa O., Monteiro A. (2013). Low genetic diversity and significant structuring in the endangered *Mentha cervina* populations and its implications for conservation. Biochem. Syst. Ecol..

[82] Jiang W., Chen X.L., Huang Y.S. (2015). Preliminary report on provenance test of *Thuja koraiensis* Nakai. Shaanxi For. Sci. Technol..

[83] Heywood V.H., Iriondo J.M. (2015). Plant conservation: Old problems, new perspectives. Biol. Conserv..

