# Supplementary material for: Genetic Evaluation of Natural Populations of the Endangered Conifer Thuja koraiensis Using Microsatellite Markers by Restriction-Associated DNA Sequencing
Source: Genes (Basel). 2018 Apr 17;9(4):218. doi: 10.3390/genes9040218 (PMC5924560; doi:10.3390/genes9040218)
Supplement: Supplementary file 1 [file genes-09-00218-s001.zip › Supplementary Files/Table S1.docx]

**Table S1.** Summary data of read using FastQC after quality control.

| **Feature** | **Value** |
| --- | --- |
| Raw Reads (M) | 39.27 |
| Raw Bases (G) | 5.867 |
| Raw Q20 (G) | 5.654(96.4%) |
| Raw Q30 (G) | 5.392(91.9%) |
| Clean Reads (M) | 36.476(92.9%) |
| Clean Bases (G) | 5.348(91.2%) |
| Clean Q20 (G) | 5.276(98.7%) |
| Clean Q30 (G) | 5.117(95.7%) |
| Average Length (bp) | 146.6 |
