# Supplementary material for: Genetic Evaluation of Natural Populations of the Endangered Conifer Thuja koraiensis Using Microsatellite Markers by Restriction-Associated DNA Sequencing
Source: Genes (Basel). 2018 Apr 17;9(4):218. doi: 10.3390/genes9040218 (PMC5924560; doi:10.3390/genes9040218)
Supplement: Supplementary file 1 [file genes-09-00218-s001.zip › Supplementary Files/Table S2.docx]

**Table S2.** The Cluster of Orthologous Groups for eukaryotic complete genomes (KOG) annotation.

| **ID** | **Class_Name** | **Contig_number** |
| --- | --- | --- |
| A | RNA processing and modification | 389 |
| B | Chromatin structure and dynamics | 82 |
| C | Energy production and conversion | 601 |
| D | Cell cycle control, cell division, chromosome partitioning | 99 |
| E | Amino acid transport and metabolism | 363 |
| F | Nucleotide transport and metabolism | 71 |
| G | Carbohydrate transport and metabolism | 457 |
| H | Coenzyme transport and metabolism | 70 |
| I | Lipid transport and metabolism | 267 |
| J | Translation, ribosomal structure and biogenesis | 514 |
| K | Transcription | 374 |
| L | Replication, recombination and repair | 174 |
| M | Cell wall/membrane/envelope biogenesis | 78 |
| N | Cell motility | 2 |
| O | Posttranslational modification, protein turnover, chaperones | 852 |
| P | Inorganic ion transport and metabolism | 263 |
| Q | Secondary metabolites biosynthesis, transport and catabolism | 621 |
| R | General function prediction only | 12,138 |
| S | Function unknown | 307 |
| T | Signal transduction mechanisms | 639 |
| U | Intracellular trafficking, secretion, and vesicular transport | 274 |
| V | Defense mechanisms | 93 |
| W | Extracellular structures | 5 |
| Y | Nuclear structure | 12 |
| Z | Cytoskeleton | 171 |
