# Supplementary material for: Genetic Evaluation of Natural Populations of the Endangered Conifer Thuja koraiensis Using Microsatellite Markers by Restriction-Associated DNA Sequencing
Source: Genes (Basel). 2018 Apr 17;9(4):218. doi: 10.3390/genes9040218 (PMC5924560; doi:10.3390/genes9040218)
Supplement: Supplementary file 1 [file genes-09-00218-s001.zip › Supplementary Files/Table S4.docx]

**Table S4.** Frequencies of repeat type with repeat numbers in SSRs from *T. Koraiensis*.

| **Repeat numbers** | **Mono** | **Di** | **Tri** | **Tetra** | **Penta** | **Hexa** | **Total** | **%** |
| --- | --- | --- | --- | --- | --- | --- | --- | --- |
| 5 | 0 | 0 | 2411 | 288 | 77 | 241 | 3017 | 7.99% |
| 6 | 0 | 3426 | 991 | 83 | 30 | 74 | 4604 | 12.19% |
| 7 | 0 | 2102 | 534 | 39 | 4 | 41 | 2720 | 7.20% |
| 8 | 0 | 1616 | 284 | 21 | 4 | 27 | 1952 | 5.17% |
| 9 | 0 | 1184 | 177 | 11 | 5 | 16 | 1393 | 3.69% |
| 10 | 6945 | 847 | 138 | 11 | 5 | 3 | 7949 | 21.05% |
| 11 | 3166 | 678 | 100 | 5 | 3 | 5 | 3957 | 10.48% |
| 12 | 1892 | 554 | 85 | 3 | 2 | 4 | 2540 | 6.73% |
| 13 | 1185 | 454 | 62 | 4 | 1 | 4 | 1710 | 4.53% |
| 14 | 820 | 347 | 57 | 4 | 0 | 5 | 1233 | 3.27% |
| 15 | 612 | 312 | 45 | 4 | 1 | 3 | 977 | 2.59% |
| 16 | 467 | 337 | 26 | 0 | 1 | 4 | 835 | 2.21% |
| 17 | 354 | 256 | 33 | 0 | 0 | 1 | 644 | 1.71% |
| 18 | 255 | 226 | 25 | 0 | 0 | 1 | 507 | 1.34% |
| 19 | 190 | 220 | 25 | 1 | 0 | 2 | 438 | 1.16% |
| 20 | 117 | 219 | 24 | 0 | 0 | 1 | 361 | 0.96% |
| ＞20 | 799 | 2017 | 102 | 4 | 0 | 2 | 2924 | 7.74% |
| Total | 16802 | 14795 | 5119 | 478 | 133 | 434 | 37761 |  |
| % | 44.50% | 39.18% | 13.56% | 1.27% | 0.35% | 1.15% |  |  |
