# Supplementary material for: Genetic Evaluation of Natural Populations of the Endangered Conifer Thuja koraiensis Using Microsatellite Markers by Restriction-Associated DNA Sequencing
Source: Genes (Basel). 2018 Apr 17;9(4):218. doi: 10.3390/genes9040218 (PMC5924560; doi:10.3390/genes9040218)
Supplement: Supplementary file 1 [file genes-09-00218-s001.zip › Supplementary Files/Table S5.docx]

**Table S5.** Frequencies of different repeat motifs in SSRs.

| **Repeats** | **5** | **6** | **7** | **8** | **9** | **10** | **11** | **12** | **13** | **14** | **15** | **16** | **17** | **18** | **19** | **20** | **＞20** | **total** | **%** |
| --- | --- | --- | --- | --- | --- | --- | --- | --- | --- | --- | --- | --- | --- | --- | --- | --- | --- | --- | --- |
| A/T | - | - | - | - | - | 6292 | 2734 | 1599 | 984 | 678 | 515 | 379 | 284 | 204 | 148 | 87 | 397 | 14301 | 37.872% |
| C/G | - | - | - | - | - | 653 | 432 | 293 | 201 | 142 | 97 | 88 | 70 | 51 | 42 | 30 | 402 | 2501 | 6.623% |
| AC/GT | - | 1005 | 706 | 508 | 360 | 253 | 208 | 183 | 156 | 134 | 119 | 116 | 118 | 91 | 92 | 76 | 651 | 4776 | 12.648% |
| AG/CT | - | 674 | 368 | 308 | 243 | 138 | 118 | 98 | 99 | 69 | 80 | 83 | 71 | 62 | 83 | 84 | 942 | 3520 | 9.322% |
| AT/AT | - | 1678 | 977 | 782 | 573 | 455 | 350 | 272 | 199 | 144 | 113 | 138 | 67 | 73 | 45 | 59 | 424 | 6349 | 16.814% |
| CG/CG | - | 69 | 51 | 18 | 8 | 1 | 2 | 1 |  |  |  |  |  |  |  |  | 0 | 150 | 0.397% |
| AAC/GTT | 417 | 126 | 65 | 34 | 23 | 7 | 10 | 5 | 5 | 9 | 2 | 1 | 5 | 3 | 1 | 2 | 5 | 720 | 1.907% |
| AAG/CTT | 509 | 154 | 92 | 42 | 24 | 24 | 22 | 20 | 18 | 14 | 11 | 7 | 8 | 5 | 7 | 6 | 20 | 983 | 2.603% |
| AAT/ATT | 387 | 153 | 104 | 44 | 23 | 21 | 14 | 6 | 4 | 1 | 3 | 2 | 1 | 3 |  |  | 2 | 768 | 2.034% |
| ACC/GGT | 153 | 64 | 28 | 19 | 13 | 6 | 4 | 7 | 4 | 2 | 1 | 4 | 1 |  |  |  | 0 | 306 | 0.810% |
| ACG/CGT | 59 | 36 | 13 | 5 | 7 | 2 | 1 | 8 | 3 |  |  |  |  |  | 1 |  | 2 | 137 | 0.363% |
| ACT/AGT | 14 | 3 | 6 | 1 |  |  |  | 2 |  |  |  |  |  |  |  |  | 0 | 26 | 0.069% |
| AGC/CTG | 82 | 32 | 17 | 4 | 3 | 2 |  | 2 |  |  |  |  |  |  |  |  | 0 | 142 | 0.376% |
| AGG/CCT | 333 | 207 | 54 | 38 | 26 | 18 | 14 | 4 | 3 | 3 | 6 |  | 1 | 1 | 1 |  | 8 | 717 | 1.899% |
| ATC/ATG | 423 | 204 | 148 | 93 | 58 | 58 | 35 | 31 | 25 | 28 | 22 | 12 | 17 | 13 | 15 | 16 | 65 | 1263 | 3.345% |
| CCG/CGG | 34 | 12 | 7 | 4 |  |  |  |  |  |  |  |  |  |  |  |  | 0 | 57 | 0.151% |
| AAAC/GTTT | 4 | 1 | 1 |  |  |  |  |  |  |  |  |  |  |  |  |  | 0 | 6 | 0.016% |
| AAAG/CTTT | 15 | 2 | 2 | 3 |  | 1 |  |  |  |  |  |  |  |  |  |  | 0 | 23 | 0.061% |
| AAAT/ATTT | 91 | 27 | 5 | 4 |  | 1 |  |  |  |  |  |  |  |  |  |  | 0 | 128 | 0.339% |
| AACC/GGTT | 1 |  | 1 |  |  |  |  |  |  |  |  |  |  |  |  |  | 0 | 2 | 0.005% |
| AACG/CGTT | 1 |  |  |  |  |  |  |  |  |  |  |  |  |  |  |  | 0 | 1 | 0.003% |
| AACT/AGTT | 3 |  |  |  |  |  |  |  |  |  |  |  |  |  |  |  | 0 | 3 | 0.008% |
| AAGG/CCTT | 2 | 1 |  |  |  |  |  |  |  |  |  |  |  |  |  |  | 0 | 3 | 0.008% |
| AAGT/ACTT |  |  | 1 |  |  |  |  |  |  |  |  |  |  |  |  |  | 0 | 1 | 0.003% |
| AATC/ATTG | 6 | 1 |  | 1 |  |  |  |  |  |  |  |  |  |  |  |  | 0 | 8 | 0.021% |
| AATG/ATTC | 16 | 3 | 1 | 1 |  |  |  |  |  |  |  |  |  |  |  |  | 0 | 21 | 0.056% |
| AATT/AATT | 12 | 3 |  |  |  |  |  |  |  |  |  |  |  |  |  |  | 0 | 15 | 0.040% |
| ACAG/CTGT | 3 | 1 | 1 |  |  | 1 | 1 |  |  |  |  |  |  |  | 1 |  | 1 | 9 | 0.024% |
| ACAT/ATGT | 89 | 34 | 24 | 9 | 9 | 6 | 3 | 2 | 2 | 4 | 3 |  |  |  |  |  | 0 | 185 | 0.490% |
| ACCT/AGGT | 1 |  |  |  |  |  |  |  |  |  |  |  |  |  |  |  | 0 | 1 | 0.003% |
| ACGC/CGTG | 12 |  |  |  |  |  |  |  |  |  |  |  |  |  |  |  | 0 | 12 | 0.032% |
| ACGG/CCGT | 1 | 1 |  |  |  |  |  |  |  |  |  |  |  |  |  |  | 0 | 2 | 0.005% |
| ACTG/AGTC | 2 |  |  |  |  |  |  |  |  |  |  |  |  |  |  |  | 0 | 2 | 0.005% |
| AGAT/ATCT | 17 | 7 | 3 | 2 | 2 | 2 | 1 |  | 2 |  | 1 |  |  |  |  |  | 3 | 40 | 0.106% |
| AGCC/CTGG | 2 |  |  |  |  |  |  |  |  |  |  |  |  |  |  |  | 0 | 2 | 0.005% |
| AGGG/CCCT | 3 | 1 |  |  |  |  |  |  |  |  |  |  |  |  |  |  | 0 | 4 | 0.011% |
| ATCC/ATGG | 5 |  |  | 1 |  |  |  | 1 |  |  |  |  |  |  |  |  | 0 | 7 | 0.019% |
| ATGC/ATGC | 1 |  |  |  |  |  |  |  |  |  |  |  |  |  |  |  | 0 | 1 | 0.003% |
| CCCG/CGGG |  | 1 |  |  |  |  |  |  |  |  |  |  |  |  |  |  | 0 | 1 | 0.003% |
| CCGG/CCGG | 1 |  |  |  |  |  |  |  |  |  |  |  |  |  |  |  | 0 | 1 | 0.003% |
| AAAAG/CTTTT | 2 | 1 |  |  |  |  |  |  |  |  |  |  |  |  |  |  | 0 | 3 | 0.008% |
| AAAAT/ATTTT | 8 | 4 |  |  |  |  |  |  |  |  |  |  |  |  |  |  | 0 | 12 | 0.032% |
| AAACC/GGTTT | 1 |  |  | 1 |  |  |  |  |  |  |  |  |  |  |  |  | 0 | 2 | 0.005% |
| AAACG/CGTTT | 1 |  |  |  |  |  |  |  |  |  |  |  |  |  |  |  | 0 | 1 | 0.003% |
| AAACT/AGTTT | 6 | 1 |  |  |  |  |  |  |  |  |  |  |  |  |  |  | 0 | 7 | 0.019% |
| AAAGG/CCTTT | 1 |  |  |  |  |  |  |  |  |  |  |  |  |  |  |  | 0 | 1 | 0.003% |
| AAATC/ATTTG | 2 |  | 1 |  |  |  |  |  |  |  |  |  |  |  |  |  | 0 | 3 | 0.008% |
| AAATG/ATTTC | 1 |  |  |  |  |  |  |  |  |  |  |  |  |  |  |  | 0 | 1 | 0.003% |
| AAATT/AATTT | 2 |  |  |  |  |  |  |  |  |  |  |  |  |  |  |  | 0 | 2 | 0.005% |
| AACAT/ATGTT |  |  |  |  |  |  | 1 |  |  |  |  |  |  |  |  |  | 0 | 1 | 0.003% |
| AACCG/CGGTT | 1 |  |  |  |  | 1 |  |  |  |  |  | 1 |  |  |  |  | 0 | 3 | 0.008% |
| AACTC/AGTTG | 1 |  |  |  |  |  |  |  |  |  |  |  |  |  |  |  | 0 | 1 | 0.003% |
| AAGAC/CTTGT |  |  | 1 |  |  |  |  |  |  |  |  |  |  |  |  |  | 0 | 1 | 0.003% |
| AAGAG/CTCTT | 2 |  |  |  |  |  |  |  |  |  |  |  |  |  |  |  | 0 | 2 | 0.005% |
| AAGAT/ATCTT | 14 | 3 |  |  |  |  |  |  |  |  |  |  |  |  |  |  | 0 | 17 | 0.045% |
| AAGGG/CCCTT |  |  |  | 1 |  |  |  |  |  |  |  |  |  |  |  |  | 0 | 1 | 0.003% |
| AAGGT/ACCTT | 1 |  |  |  |  |  |  |  |  |  |  |  |  |  |  |  | 0 | 1 | 0.003% |
| AAGTG/ACTTC | 1 |  |  |  |  |  |  |  |  |  |  |  |  |  |  |  | 0 | 1 | 0.003% |
| AATAG/ATTCT | 2 |  |  |  |  |  |  |  |  |  |  |  |  |  |  |  | 0 | 2 | 0.005% |
| AATAT/ATATT | 8 | 2 |  |  | 1 | 1 |  |  |  |  |  |  |  |  |  |  | 0 | 12 | 0.032% |
| AATCC/ATTGG | 1 |  |  |  |  |  |  |  |  |  |  |  |  |  |  |  | 0 | 1 | 0.003% |
| AATGC/ATTGC | 1 |  |  |  |  |  |  |  |  |  |  |  |  |  |  |  | 0 | 1 | 0.003% |
| AATGG/ATTCC | 1 |  |  |  | 1 |  |  |  |  |  |  |  |  |  |  |  | 0 | 2 | 0.005% |
| ACACC/GGTGT | 1 |  |  |  |  |  |  |  |  |  |  |  |  |  |  |  | 0 | 1 | 0.003% |
| ACAGC/CTGTG | 2 |  |  |  |  |  |  |  |  |  |  |  |  |  |  |  | 0 | 2 | 0.005% |
| ACATC/ATGTG | 1 |  |  |  |  |  |  |  |  |  |  |  |  |  |  |  | 0 | 1 | 0.003% |
| ACCAG/CTGGT | 1 |  |  |  |  |  |  |  |  |  |  |  |  |  |  |  | 0 | 1 | 0.003% |
| ACCAT/ATGGT | 1 |  |  |  |  |  |  |  |  |  |  |  |  |  |  |  | 0 | 1 | 0.003% |
| ACCCT/AGGGT | 6 | 13 |  |  |  |  |  | 1 | 1 |  | 1 |  |  |  |  |  | 0 | 22 | 0.058% |
| ACCGC/CGGTG |  |  |  |  |  |  |  | 1 |  |  |  |  |  |  |  |  | 0 | 1 | 0.003% |
| ACCTC/AGGTG |  | 1 |  |  |  |  |  |  |  |  |  |  |  |  |  |  | 0 | 1 | 0.003% |
| ACTCG/AGTCG | 1 |  |  |  |  |  |  |  |  |  |  |  |  |  |  |  | 0 | 1 | 0.003% |
| AGAGC/CTCTG |  | 1 |  |  |  |  |  |  |  |  |  |  |  |  |  |  | 0 | 1 | 0.003% |
| AGAGG/CCTCT | 1 |  |  |  |  |  |  |  |  |  |  |  |  |  |  |  | 0 | 1 | 0.003% |
| AGATG/ATCTC | 1 |  |  |  |  |  |  |  |  |  |  |  |  |  |  |  | 0 | 1 | 0.003% |
| AGCCG/CGGCT |  |  |  |  |  |  | 1 |  |  |  |  |  |  |  |  |  | 0 | 1 | 0.003% |
| AGCCT/AGGCT | 1 |  |  |  |  |  |  |  |  |  |  |  |  |  |  |  | 0 | 1 | 0.003% |
| AGCGG/CCGCT | 1 |  | 2 |  |  |  |  |  |  |  |  |  |  |  |  |  | 0 | 3 | 0.008% |
| AGGGG/CCCCT | 2 | 2 |  |  |  |  |  |  |  |  |  |  |  |  |  |  | 0 | 4 | 0.011% |
| ATCCC/ATGGG |  | 2 |  | 2 | 2 | 3 | 1 |  |  |  |  |  |  |  |  |  | 0 | 10 | 0.026% |
| ATCGC/ATGCG | 1 |  |  |  |  |  |  |  |  |  |  |  |  |  |  |  | 0 | 1 | 0.003% |
| ATGCC/ATGGC |  |  |  |  | 1 |  |  |  |  |  |  |  |  |  |  |  | 0 | 1 | 0.003% |
| AAAAAC/GTTTTT |  |  | 1 |  |  |  |  |  |  |  |  |  |  |  |  |  | 0 | 1 | 0.003% |
| AAAAAG/CTTTTT | 11 | 2 | 2 |  |  |  |  |  |  |  |  |  |  |  |  |  | 0 | 15 | 0.040% |
| AAAAAT/ATTTTT | 3 | 1 |  |  |  |  |  |  |  |  |  |  |  |  |  |  | 0 | 4 | 0.011% |
| AAACAG/CTGTTT | 1 |  |  |  |  |  |  |  |  |  |  |  |  |  |  |  | 0 | 1 | 0.003% |
| AAACAT/ATGTTT | 1 |  |  |  |  |  |  |  |  |  |  |  |  |  |  |  | 0 | 1 | 0.003% |
| AAAGAG/CTCTTT |  |  | 1 |  |  |  |  |  |  |  |  |  |  |  |  |  | 0 | 1 | 0.003% |
| AAAGGG/CCCTTT | 4 |  |  |  |  |  |  |  |  |  |  |  |  |  |  |  | 0 | 4 | 0.011% |
| AAAGTC/ACTTTG |  |  | 1 |  |  |  |  |  |  |  |  |  |  |  |  |  | 0 | 1 | 0.003% |
| AAATCG/ATTTCG |  | 1 |  |  |  |  |  |  |  |  |  |  |  |  |  |  | 0 | 1 | 0.003% |
| AAATCT/AGATTT | 1 |  |  |  |  |  |  |  |  |  |  |  |  |  |  |  | 0 | 1 | 0.003% |
| AACAAG/CTTGTT | 2 | 1 |  | 1 |  |  |  |  |  |  |  |  |  |  |  |  | 0 | 4 | 0.011% |
| AACACC/GGTGTT | 1 |  |  |  | 1 |  |  |  |  |  | 1 |  |  |  |  |  | 0 | 3 | 0.008% |
| AACAGC/CTGTTG |  |  |  | 2 |  |  |  |  |  | 1 | 1 |  |  |  |  |  | 0 | 4 | 0.011% |
| AACATT/AATGTT | 1 |  |  |  |  |  |  |  |  |  |  |  |  |  |  |  | 0 | 1 | 0.003% |
| AACCCC/GGGGTT | 1 |  |  |  |  |  |  |  |  |  |  |  |  |  |  |  | 0 | 1 | 0.003% |
| AACCCG/CGGGTT | 3 | 1 |  |  |  |  |  |  |  |  |  |  |  |  |  |  | 0 | 4 | 0.011% |
| AACCCT/AGGGTT | 18 | 4 | 4 | 1 |  |  |  |  |  |  |  |  |  |  |  |  | 0 | 27 | 0.072% |
| AACCGG/CCGGTT | 1 |  |  |  |  |  |  |  |  |  |  |  |  |  |  |  | 0 | 1 | 0.003% |
| AACCTG/AGGTTC | 1 |  |  |  |  |  |  |  |  |  |  |  |  |  |  |  | 0 | 1 | 0.003% |
| AACGAC/CGTTGT |  |  |  | 1 |  |  |  |  |  |  |  |  |  |  |  |  | 0 | 1 | 0.003% |
| AACTAC/AGTTGT | 2 |  |  |  |  |  |  |  |  |  |  |  |  |  |  |  | 0 | 2 | 0.005% |
| AACTCG/AGTTCG | 1 |  |  |  |  |  |  |  |  |  |  |  |  |  |  |  | 0 | 1 | 0.003% |
| AACTCT/AGAGTT | 1 |  |  |  |  |  |  |  |  |  |  |  |  |  |  |  | 0 | 1 | 0.003% |
| AAGAGG/CCTCTT | 6 | 1 | 1 | 2 |  |  |  |  |  |  |  |  |  |  |  |  | 0 | 10 | 0.026% |
| AAGATC/ATCTTG |  | 1 |  |  |  |  |  |  |  |  |  |  |  |  |  |  | 0 | 1 | 0.003% |
| AAGCAG/CTGCTT | 1 |  |  |  |  |  |  |  |  |  |  |  |  |  |  |  | 0 | 1 | 0.003% |
| AAGCTC/AGCTTG | 1 |  |  |  |  |  |  |  |  |  |  |  |  |  |  |  | 0 | 1 | 0.003% |
| AAGGAG/CCTTCT | 4 | 5 | 2 | 2 |  |  | 1 |  |  |  |  |  |  |  |  |  | 0 | 14 | 0.037% |
| AAGGGC/CCCTTG | 1 |  |  |  |  |  |  |  |  |  |  |  |  |  |  |  | 0 | 1 | 0.003% |
| AAGGGG/CCCCTT | 2 |  |  |  |  |  |  |  |  |  |  |  |  |  |  |  | 0 | 2 | 0.005% |
| AAGGGT/ACCCTT | 7 |  |  |  |  |  |  |  |  |  |  |  |  |  |  |  | 0 | 7 | 0.019% |
| AAGTGG/ACTTCC |  | 2 |  |  |  |  |  |  |  |  |  |  |  |  |  |  | 0 | 2 | 0.005% |
| AATATT/AATATT | 1 |  |  |  |  |  |  |  |  |  |  |  |  |  |  |  | 0 | 1 | 0.003% |
| AATCAG/ATTCTG | 1 |  |  |  |  |  |  |  |  |  |  |  |  |  |  |  | 0 | 1 | 0.003% |
| AATCCT/AGGATT | 1 |  |  |  |  |  |  |  |  |  |  |  |  |  |  |  | 0 | 1 | 0.003% |
| AATCTC/AGATTG |  | 1 |  |  |  |  |  |  |  |  |  |  |  |  |  |  | 0 | 1 | 0.003% |
| AATGAC/ATTGTC | 1 |  |  |  |  |  |  |  |  |  |  |  |  |  |  |  | 0 | 1 | 0.003% |
| AATGAG/ATTCTC | 2 |  |  |  |  |  |  |  |  |  |  |  |  |  |  |  | 0 | 2 | 0.005% |
| AATGGG/ATTCCC |  | 1 |  |  |  |  |  |  |  |  |  |  |  |  |  |  | 0 | 1 | 0.003% |
| AATTCC/AATTGG | 1 |  |  |  |  |  |  |  |  |  |  |  |  |  |  |  | 0 | 1 | 0.003% |
| ACACAG/CTGTGT | 4 |  |  |  |  |  |  |  |  |  |  |  |  |  |  |  | 0 | 4 | 0.011% |
| ACACAT/ATGTGT | 8 | 4 | 4 | 1 | 3 |  |  | 2 |  | 2 | 1 | 3 |  | 1 | 1 | 1 | 2 | 33 | 0.087% |
| ACACCC/GGGTGT | 3 | 2 |  | 1 |  |  |  |  |  |  |  |  |  |  |  |  | 0 | 6 | 0.016% |
| ACACCG/CGGTGT | 1 | 2 |  |  |  |  |  |  |  |  |  |  |  |  |  |  | 0 | 3 | 0.008% |
| ACACGC/CGTGTG | 4 |  | 1 |  | 1 | 1 | 1 |  |  |  |  |  |  |  |  |  | 0 | 8 | 0.021% |
| ACACTC/AGTGTG |  |  | 1 |  |  |  |  |  |  |  |  |  |  |  |  |  | 0 | 1 | 0.003% |
| ACAGAT/ATCTGT |  | 1 |  |  |  |  |  |  |  |  |  |  |  |  |  |  | 0 | 1 | 0.003% |
| ACAGCC/CTGTGG | 1 |  |  |  |  |  |  |  |  |  |  |  |  |  |  |  | 0 | 1 | 0.003% |
| ACAGGC/CCTGTG | 1 |  |  |  |  |  |  |  |  |  |  |  |  |  |  |  | 0 | 1 | 0.003% |
| ACATAG/ATGTCT |  | 1 |  |  | 1 |  |  |  |  |  |  |  |  |  |  |  | 0 | 2 | 0.005% |
| ACATAT/ATATGT | 25 | 9 | 6 | 4 | 2 | 2 | 2 |  | 2 | 1 |  |  |  |  |  |  | 0 | 53 | 0.140% |
| ACATCC/ATGTGG | 1 |  |  |  |  |  |  |  |  |  |  |  |  |  |  |  | 0 | 1 | 0.003% |
| ACATGC/ATGTGC | 3 |  |  |  |  |  |  |  |  |  |  |  |  |  |  |  | 0 | 3 | 0.008% |
| ACCACT/AGTGGT | 5 |  | 1 |  |  |  |  |  |  |  |  |  |  |  |  |  | 0 | 6 | 0.016% |
| ACCAGC/CTGGTG | 2 |  |  |  |  |  |  |  |  | 1 |  |  |  |  |  |  | 0 | 3 | 0.008% |
| ACCATC/ATGGTG | 8 | 6 | 7 | 6 | 1 |  |  |  |  |  |  |  | 1 |  |  |  | 0 | 29 | 0.077% |
| ACCCCC/GGGGGT | 1 |  |  |  |  |  |  |  |  |  |  |  |  |  |  |  | 0 | 1 | 0.003% |
| ACCCCG/CGGGGT |  | 1 |  |  |  |  |  |  |  |  |  |  |  |  |  |  | 0 | 1 | 0.003% |
| ACCCCT/AGGGGT | 14 | 3 |  |  |  |  |  |  |  |  |  |  |  |  |  |  | 0 | 17 | 0.045% |
| ACCGAT/ATCGGT |  | 1 |  |  |  |  |  |  |  |  |  |  |  |  |  |  | 0 | 1 | 0.003% |
| ACCGCC/CGGTGG | 1 | 1 | 1 |  |  |  |  |  |  |  |  |  |  |  |  |  | 0 | 3 | 0.008% |
| ACCGGT/ACCGGT |  | 1 |  |  |  |  |  |  |  |  |  |  |  |  |  |  | 0 | 1 | 0.003% |
| ACCGTC/ACGGTG | 1 |  |  |  |  |  |  |  |  |  |  |  |  |  |  |  | 0 | 1 | 0.003% |
| ACCTAT/AGGTAT |  |  | 1 |  |  |  |  |  |  |  |  |  |  |  |  |  | 0 | 1 | 0.003% |
| ACCTCC/AGGTGG | 7 | 2 | 2 |  |  |  |  |  |  |  |  |  |  |  |  |  | 0 | 11 | 0.029% |
| ACCTCT/AGAGGT | 2 |  |  |  |  |  |  |  |  |  |  |  |  |  |  |  | 0 | 2 | 0.005% |
| ACGAGG/CCTCGT | 1 |  |  |  |  |  |  |  |  |  |  |  |  |  |  |  | 0 | 1 | 0.003% |
| ACGATG/ATCGTC | 12 | 5 | 3 | 2 | 6 |  | 1 |  | 2 |  |  | 1 |  |  | 1 |  | 0 | 33 | 0.087% |
| ACGGGG/CCCCGT | 2 | 1 |  |  |  |  |  |  |  |  |  |  |  |  |  |  | 0 | 3 | 0.008% |
| ACTAGC/AGTGCT |  | 1 |  |  |  |  |  |  |  |  |  |  |  |  |  |  | 0 | 1 | 0.003% |
| ACTATC/AGTGAT | 4 |  |  |  |  |  |  |  |  |  |  |  |  |  |  |  | 0 | 4 | 0.011% |
| ACTCCC/AGTGGG | 1 | 1 |  |  |  |  |  |  |  |  |  |  |  |  |  |  | 0 | 2 | 0.005% |
| ACTCGG/AGTCCG | 1 |  |  |  |  |  |  |  |  |  |  |  |  |  |  |  | 0 | 1 | 0.003% |
| ACTGAT/AGTATC | 2 |  | 1 |  |  |  |  |  |  |  |  |  |  |  |  |  | 0 | 3 | 0.008% |
| ACTGCG/AGTCGC | 2 |  |  |  |  |  |  |  |  |  |  |  |  |  |  |  | 0 | 2 | 0.005% |
| AGAGGC/CCTCTG | 3 |  |  |  |  |  |  |  |  |  |  |  |  |  |  |  | 0 | 3 | 0.008% |
| AGAGGG/CCCTCT | 4 | 5 |  |  |  |  |  |  |  |  |  |  |  |  |  |  | 0 | 9 | 0.024% |
| AGATAT/ATATCT | 2 |  |  |  |  |  |  |  |  |  |  |  |  |  |  |  | 0 | 2 | 0.005% |
| AGATGG/ATCTCC | 1 |  |  |  |  |  |  |  |  |  |  |  |  |  |  |  | 0 | 1 | 0.003% |
| AGCAGG/CCTGCT | 1 |  |  |  |  |  |  |  |  |  |  |  |  |  |  |  | 0 | 1 | 0.003% |
| AGCATC/ATGCTG | 2 |  |  |  |  |  |  |  |  |  |  |  |  |  |  |  | 0 | 2 | 0.005% |
| AGCATG/ATGCTC |  |  |  |  |  |  |  | 1 |  |  |  |  |  |  |  |  | 0 | 1 | 0.003% |
| AGCCAT/ATGGCT | 12 | 1 | 1 | 4 |  |  |  |  |  |  |  |  |  |  |  |  | 0 | 18 | 0.048% |
| AGCCGC/CGGCTG | 1 |  |  |  |  |  |  |  |  |  |  |  |  |  |  |  | 0 | 1 | 0.003% |
| AGCCTC/AGGCTG | 1 |  |  |  |  |  |  |  |  |  |  |  |  |  |  |  | 0 | 1 | 0.003% |
| AGCCTG/AGGCTC | 1 | 1 |  |  |  |  |  |  |  |  |  |  |  |  |  |  | 0 | 2 | 0.005% |
| AGGATG/ATCCTC | 3 | 2 |  |  |  |  |  | 1 |  |  |  |  |  |  |  |  | 0 | 6 | 0.016% |
| AGGCAT/ATGCCT | 2 |  |  |  |  |  |  |  |  |  |  |  |  |  |  |  | 0 | 2 | 0.005% |
| AGGCGG/CCGCCT |  | 1 |  |  |  |  |  |  |  |  |  |  |  |  |  |  | 0 | 1 | 0.003% |
| AGGGAT/ATCCCT | 2 |  |  |  |  |  |  |  |  |  |  |  |  |  |  |  | 0 | 2 | 0.005% |
| AGGGCG/CCCTCG | 1 |  |  |  |  |  |  |  |  |  |  |  |  |  |  |  | 0 | 1 | 0.003% |
| ATCGCC/ATGGCG | 3 |  |  |  | 1 |  |  |  |  |  |  |  |  |  |  |  | 0 | 4 | 0.011% |
| ATGCCC/ATGGGC | 3 |  |  |  |  |  |  |  |  |  |  |  |  |  |  |  | 0 | 3 | 0.008% |
| CCCCCG/CGGGGG |  | 1 |  |  |  |  |  |  |  |  |  |  |  |  |  |  | 0 | 1 | 0.003% |
| total | 3017 | 4604 | 2720 | 1952 | 1393 | 7949 | 3957 | 2540 | 1710 | 1233 | 977 | 835 | 644 | 507 | 438 | 361 | 2924 | 37761 | 100.000% |
