# Supplementary material for: Genetic Evaluation of Natural Populations of the Endangered Conifer Thuja koraiensis Using Microsatellite Markers by Restriction-Associated DNA Sequencing
Source: Genes (Basel). 2018 Apr 17;9(4):218. doi: 10.3390/genes9040218 (PMC5924560; doi:10.3390/genes9040218)
Supplement: Supplementary file 1 [file genes-09-00218-s001.zip › Supplementary Files/Table S7.docx]

**Table S7.** Nei's genetic distance (below diagonal) and genetic identity (above diagonal) among the three populations.

| **Populations** | **LGZ** | **SDG** | **DJG** |
| --- | --- | --- | --- |
| LGZ |  | 0.821 | 0.874 |
| SDG | 0.197 |  | 0.792 |
| DJG | 0.135 | 0.233 |  |
